# AMPA receptor deletion in developing MGE-derived hippocampal interneurons causes a redistribution of excitatory synapses and attenuates postnatal network oscillatory activity

**DOI:** 10.1038/s41598-020-58068-6

**Published:** 2020-01-28

**Authors:** Gülcan Akgül, Chris J. McBain

**Affiliations:** 1 Porter Neuroscience Research Centre, Rm3C903, Lincoln Drive, Eunice Kennedy Shriver National Institute of Child Health and Human Development, Bethesda, MD 20892 USA; 2 Present Address: Department of Physiology and Neurobiology, University of Connecticut, 75 N Eagleville Rd., Storrs, CT 06269 USA

**Keywords:** Neuroscience, Cellular neuroscience, Ion channels in the nervous system

## Abstract

Inhibitory interneurons derived from the medial ganglionic eminence represent the largest cohort of GABAergic neurons in the hippocampus. In the CA1 hippocampus excitatory synapses onto these cells comprise GluA2-lacking, calcium-permeable AMPARs. Although synaptic transmission is not established until early in their postnatal life, AMPARs are expressed early in development, however their role is enigmatic. Using the Nkx2.1-cre mouse line we genetically deleted GluA1, GluA2, GluA3 selectively from MGE derived interneurons early in development. We observed that the number of MGE-derived interneurons was preserved in mature hippocampus despite early elimination of AMPARs, which resulted in >90% decrease in spontaneous excitatory synaptic activity. Of particular interest, excitatory synaptic sites were shifted from dendritic to somatic locations while maintaining a normal NMDAR content. The developmental switch of NMDARs from GluN2B-containing early in development to GluN2A-containing on maturation was similarly unperturbed despite the loss of AMPARs. Early network giant depolarizing potential oscillatory activity was compromised in early postnatal days as was both feedforward and feedback inhibition onto pyramidal neurons underscoring the importance of glutamatergic drive onto MGE-derived interneurons for hippocampal circuit function.

## Introduction

GABAergic inhibitory interneurons destined to populate neuronal circuits of hippocampus and cortex proliferate from neuronal progenitors in the medial (MGE) and caudal (CGE) ganglionic eminences of the embryonic telencephalon. Nkx2.1-positive MGE progenitors give rise to the vast majority of parvalbumin- (PV), somatostatin- (SOM) and nNOS-containing Ivy and neurogliaform interneurons^1,2^. Although these interneuron subpopulations often occupy distinct anatomical subregions within the hippocampus and target different domains of their downstream targets all MGE-derived interneurons in the CA1 hippocampus typically express synaptic GluA2-lacking, Ca^2+^-permeable AMPARs (CP-AMPARs)^3^. The majority of AMPARs on MGE-derived interneurons are comprised of GluA1 homomers^1,4^, however in PV-containing interneurons GluA4 expression commences during the early postnatal period (P14-21) converting GluA1 homomers into GluA1/4 heteromers endowing synaptic receptors with rapid kinetics^1,3,5^. Unlike principal neurons which receive excitatory synaptic input only onto dendritic spines, excitatory synapses are found on both soma and dendrites of inhibitory interneurons^6^. However, it is unclear whether these somatic versus dendritic receptors play distinct roles or are targeted by differing afferents. Although the roles of AMPARs at excitatory synapses onto inhibitory interneurons have been well documented^1,3,5^, their expression prior to synaptogenesis suggests additional roles for them in proliferation, migration and circuit development.

Migrating interneurons in the cortical plate express both AMPAR and NMDARs. Indeed GluA1–3, and GluN1/GluN2B subunits are expressed as early as embryonic stages E14 and E17 respectively in developing cortical tissue^7,8^. The role(s) of these early expressed glutamate receptors is unclear but glutamate, primarily acting through AMPA receptors, is a known chemoattractant and considered important for cortical interneuronal migration^9,10^. Block of AMPAR activation in migrating interneurons impacts the elaboration of cell processes in neocortex^11^. In cortico-hippocampal explants made from GAD67–EGFP knock-in embryos, pharmacological block of Ca^2+^-permeable AMPARs prevented migration of GAD67–EGFP interneurons into the hippocampus^10^. However, in our previous study, embryonic deletion of AMPARs from the 5HT3aR-positive, CGE-derived hippocampal interneuron cohort did not alter the overall numbers of cells (although the proportion of the CCK-containing subgroup increased and the VIP-containing subgroup decreased), it did cause an enlargement of the dendritic tree concomitant with dendritic synapse loss^12^. The impact of elimination of Gria1-3 in the MGE-derived interneuron population remains unexplored.

In this study, we investigate the role of AMPARs in the maturation of MGE-derived interneurons in hippocampus and the functional impact of reduced MGE-interneuron excitatory recruitment on hippocampal microcircuits through development. We genetically deleted Gria1-3 floxed with loxP cassettes under the control of the homeobox transcription factor, Nkx2.1 expression (Nkx2.1-Cre background). Loss of AMPARs caused near elimination of AMPAR-mediated spontaneous excitatory postsynaptic currents (sEPSCs) commencing in the early postnatal period, which was accompanied by a change in the release probability of evoked transmission. The synchronized activity of developing hippocampal circuits in neonatal animals was attenuated, as was recruitment of both feedforward and feedback inhibitory drive onto pyramidal cells. Although cell numbers were largely unchanged we observed a major redistribution of excitatory synapses away from dendritic sites onto the somatic compartment.

## Results

### MGE-derived interneuron number is not regulated by loss of AMPARs

**Figure 1:**
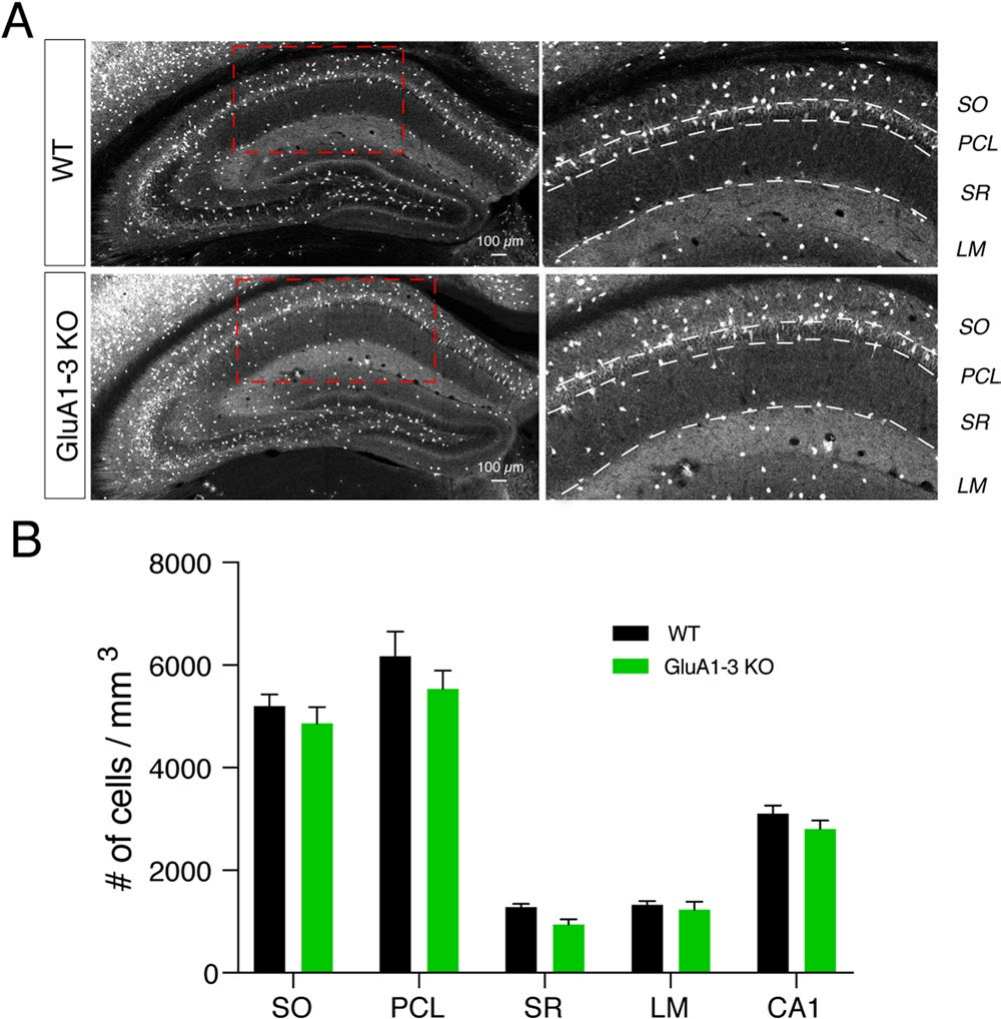
The number of MGE interneurons in CA1 hippocampus remains unchanged following AMPAR deletion. Low and high resolution confocal images of hippocampi from WT and KO mouse brains show that MGE interneurons are segregated to sublayers (SO, PCL, SR, SLM) of hippocampi comparably in both lines (**A**). (**B**) Bar graph shows no significant change in total number of MGE-derived interneurons in neither the entire CA1 subfield nor any particular subfield in KO. Tissue was collected from 4–6 sections from 5 WT (black) and 5 KO (green) mouse brains.

**Figure 2:**
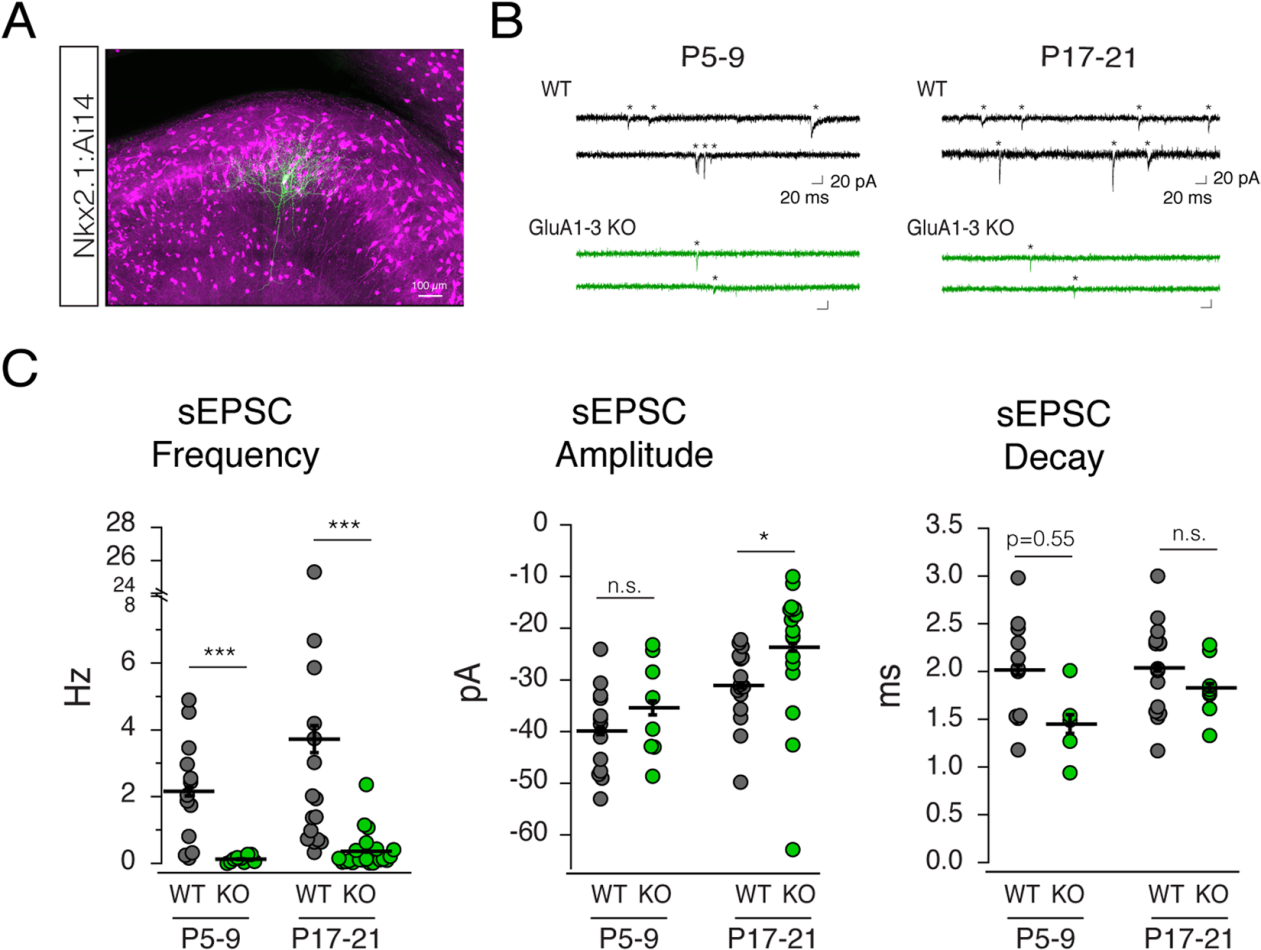
Spontaneous excitatory input onto MGE-derived interneurons is markedly decreased following GluA1-3 loss. (**A**) Confocal image of CA1 hippocampus in Nkx2.1:Ai14 mouse brain. Red tdTomato signal signifies MGE-derived interneurons and the green (streptavidin-Alexa 488) single neuron is a biocytin filled interneuron recovered following electrophysiological recording. (**B**) Representative spontaneous EPSCs (sEPSCs) recorded from MGE-derived interneurons of CA1 hippocampi of P5-9 and P17-21 WT (Nkx2.1:Ai14) or GluA1-3 KO (Nkx2.1:Ai14:Gria1-3^fl/fl^) mice. *Mark individual synaptic events. Recordings were done in the presence of picrotoxin (50 μM). (**C**) Group data are graphed for the sEPSC frequency, amplitude, decay. *P < 0.05, ***P < 0.0005.

We first determined whether the loss of function of GluA1, 2 and 3 AMPAR subunits impacted the number of MGE-derived, Nkx2.1-lineage interneurons observed in mature hippocampus. To eliminate AMPARs in MGE interneurons during embryogenesis, we crossed the Nkx2.1-Cre line with a transgenic line in which three AMPAR subunit expressing genes (*Gria1-3*), were flanked by lox cassettes (Gria1-3^f/f^) to generate offspring that were homozygous knockout for all three genes. To label and visualize MGE interneurons, we also introduced Cre/lox dependent tdTomato expression by including Ai14 (Figs. 1 and 2A). In agreement with previous reports^2,3,13^, MGE interneurons highly populate *stratum oriens* (SO) and *stratum pyramidale* (PCL) and are also present throughout *stratum radiatum* (SR) and *stratum lacunosum moleculare* (LM) of CA1 (Figs. 1A and 2). Confocal images were captured of hippocampi from 4–6 sections of each P20-21 WT (n = 5) and KO (n = 5) mouse brains (Fig. 1A). Cell quantification (Fig. 1B) showed that the total Nkx2.1-lineage, MGE-derived interneuron number within the CA1 subfield remained comparable between WT and KO hippocampus CA1 (WT vs KO: 3103 ± 159.6 vs 2807 ± 165.8 cells/mm^3^, P = 0.23). Across the CA1 subfield (LM, SR, PCL and SO) cell numbers were also unchanged from WT (LM: 1328 ± 75.3 vs 1229 ± 159.2 cell/mm^3^, P = 0.85; SR: 1283 ± 63.9 vs 946 ± 100.9, P = 0.35; PCL: 6171 ± 488.2 vs 5538 ± 352.7 cell/mm^3^, P = 0.88; SO: 5201 ± 228.1 vs 4862 ± 320.2 cell/mm^3^, P = 0.99; Two way ANOVA, F_layer_(4,40) = 150.2 P < 0.0001, F_genotype_(1,40) = 4.72 P = 0.04, F_interaction_(4,40) = 0.3 P = 0.88; followed by Sidak’s multiple comparisons) (Fig. 1A,B). These data indicate that embryonic deletion of AMPARs does not impact the overall numbers of MGE-derived interneurons that populate the mature CA1 hippocampus.

### AMPARs regulate both post- and pre-synaptic properties of excitatory synapses onto MGE-derived hippocampal interneuron

To characterize AMPAR-mediated excitatory synaptic transmission in the GluA1-3 knock-out (KO), we targeted tdTomato expressing MGE-derived interneurons in the SR and PCL of CA1 hippocampus for electrophysiological interrogation (Fig. 2A). Excitatory synaptic transmission onto MGE-derived interneurons commences around P5 and reaches maturation by ~P17^3,14^. Using wild type (WT, Nkx2.1:Ai14) and GluA1-3 knockout (MGE GluA1-3 KO, Nkx2.1:Ai14:Gria1-3^f/f^) mice at two developmental age groups (P5-9 and P17-21; Fig. 2B,C) we first recorded spontaneous excitatory postsynaptic currents (sEPSCs) in voltage clamp mode at −70 mV (in the presence of the GABA_A_ receptor antagonist picrotoxin). In both P5-9 and P17-21 KO interneurons the sEPSC frequency was reduced by > 90% compared to WT (P5-9: WT, 2.15 ± 0.16 Hz, n = 13 cells from 6 animals; KO, 0.12 ± 0.01 Hz, n = 10 cells from 3 animals; P < 0.0001; P17-21: WT, 3.72 ± 0.68 Hz, n = 16 cells from 9 animals; KO, 0.37 ± 0.06 Hz, n = 21 cells from 5 animals; P < 0.0001, Mann-Whitney Test) (Fig. 2B,C). Despite the large reduction in sEPSC frequency at both ages tested the amplitude of those events remaining was unchanged at P5-9 (WT, −39.9 ± 0.89 pA, n = 13 cells from 6 animals; KO, −35.4 ± 1.27, n = 8 cells from 2 animals; P = 0.66, Mann-Whitney Test; Fig. 2C), but was reduced by 24% by P17-21 (WT, −31.1 ± 0.82 pA, n = 16 cells from 9 animals; KO, −23.7 ± 1.35, n = 18 cells from 5 animals; P = 0.004, Mann-Whitney Test; Fig. 2C).

### Synaptic sites are redistributed toward somatic locations following AMPAR elimination

**Figure 3:**
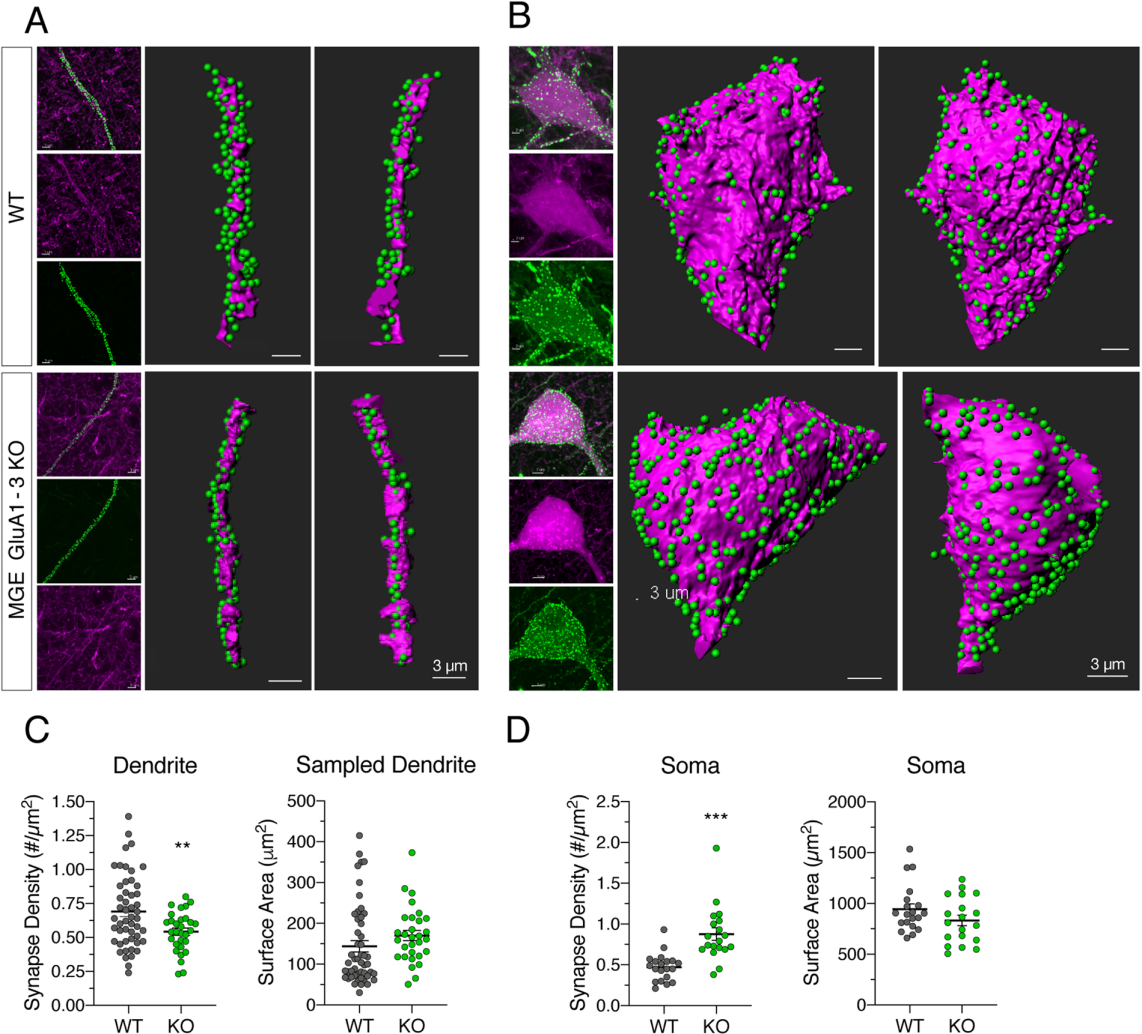
Loss of GluA1-3 shifts subcellular synaptic localization toward somatic sites Imaris reconstructions of PSD95-egfp expressing excitatory synapses (green fluorescent puncta) on MGE-derived interneuron dendrites running through SR of CA1 (red tdTomato fluorescence) (**A**) and soma located in PCL and SR of CA1 (red tdTomato fluorescence) (**B**) of WT and GluA1-3 KO MGE interneurons. Soma and dendrites were imaged using a Zeiss Airy LSM880 and quantified and graphed (C-D). A significant shift in subcellular localization of excitatory synapses, from dendrites (**C**) towards soma (**D**), was observed in MGE KO interneurons. Synapse density: WT_soma_, 0.47 ± 0.04/µm^2^, n = 20 soma from 2 animals; KO_soma_, 0.88 ± 0.08/µm^2^, n = 19 soma from 2 animals, P < 0.0001; WT_dendrite_, 0.69 ± 0.04/µm^2^, n = 50 dendritic segments from 2 animals; KO_dendrite_, 0.54 ± 0.03/µm^2^, n = 30 dendritic segments from 2 animals, P = 0.0019. Soma size, WT: 945 ± 53 µm^2^, n = 20 soma from 2 animals; KO: 833 ± 0.03 µm^2^, n = 19 soma from 2 animals; P = 0.14. Dendrite surface size sampled, WT: 144 ± 14 µm^2^, n = 50 dendritic segments from 2 animals; KO: 170 ± 12 µm^2^, n = 30 dendritic segments from 2 animals; P = 0.16. Unpaired t test with Welch’s correction, two-tailed. *P < 0.05, ***P < 0.005.

PSD-95, an essential postsynaptic component of excitatory synapses, is widely expressed in PV and SOM interneurons through postnatal development^15^. To determine whether elimination of AMPAR subunits influences the location of excitatory synaptic sites on MGE interneurons, we stereotaxically injected an adenoassociated virus (AAV) carrying Cre-dependent, fluorescently tagged PSD-95 (PSD-95-EGFP) (Fig. 3) into WT (n = 2) and MGE GluA1-3 KO (n = 2) hippocampus. Confocal images were taken of randomly selected representative dendrites (WT, n = 20; KO, n = 19) (Fig. 3A) and soma (WT, n = 50; KO, n = 30) (Fig. 3B) of MGE interneurons in CA1 hippocampus. Analysis of EGFP fluorescence puncta on defined WT and KO dendrites revealed a 22% decrease in excitatory synapse density on KO MGE interneuron dendrites (0.54 ± 0.03/µm^2^) compared to WT (0.69 ± 0.04 /µm^2^; P = 0.0019; t test with Welch’s correction, two-tailed) (Fig. 3C,D). In contrast, there was an 86% increase in the number of PSD-95 positive puncta identified on the soma of KO MGE interneurons (0.88 ± 0.08/µm^2^) versus WT MGE interneurons (0.47 ± 0.04/µm^2^; P < 0.0001; t test with Welch’s correction, two-tailed) (Fig. 3C,D). Importantly, the average surface area of the soma across groups was comparable indicating that overall soma size remained the same in KO (KO, 833 ± 5 2 µm^2^; WT, 945 ± 53 µm^2^; P = 0.14; t test with Welch’s correction, two-tailed) (Fig. 3B,D). Similarly, the average area of dendrites sampled for both WT and KO MGE interneurons were not significantly different (KO, 170 ± 12 µm^2^; WT, 144 ± 14 µm^2^; P = 0.16; t test with Welch’s correction, two-tailed) (Fig. 3A,C). These results suggest that GluA1-3 subunits of AMPARs are important determinants of subcellular synaptic localization during development and that their elimination during early development results in a shift from dendritic to more somatic locations.

### Early network driven Giant Depolarizing Currents rely on AMPAR-mediated recruitment of MGE interneurons

**Figure 4:**
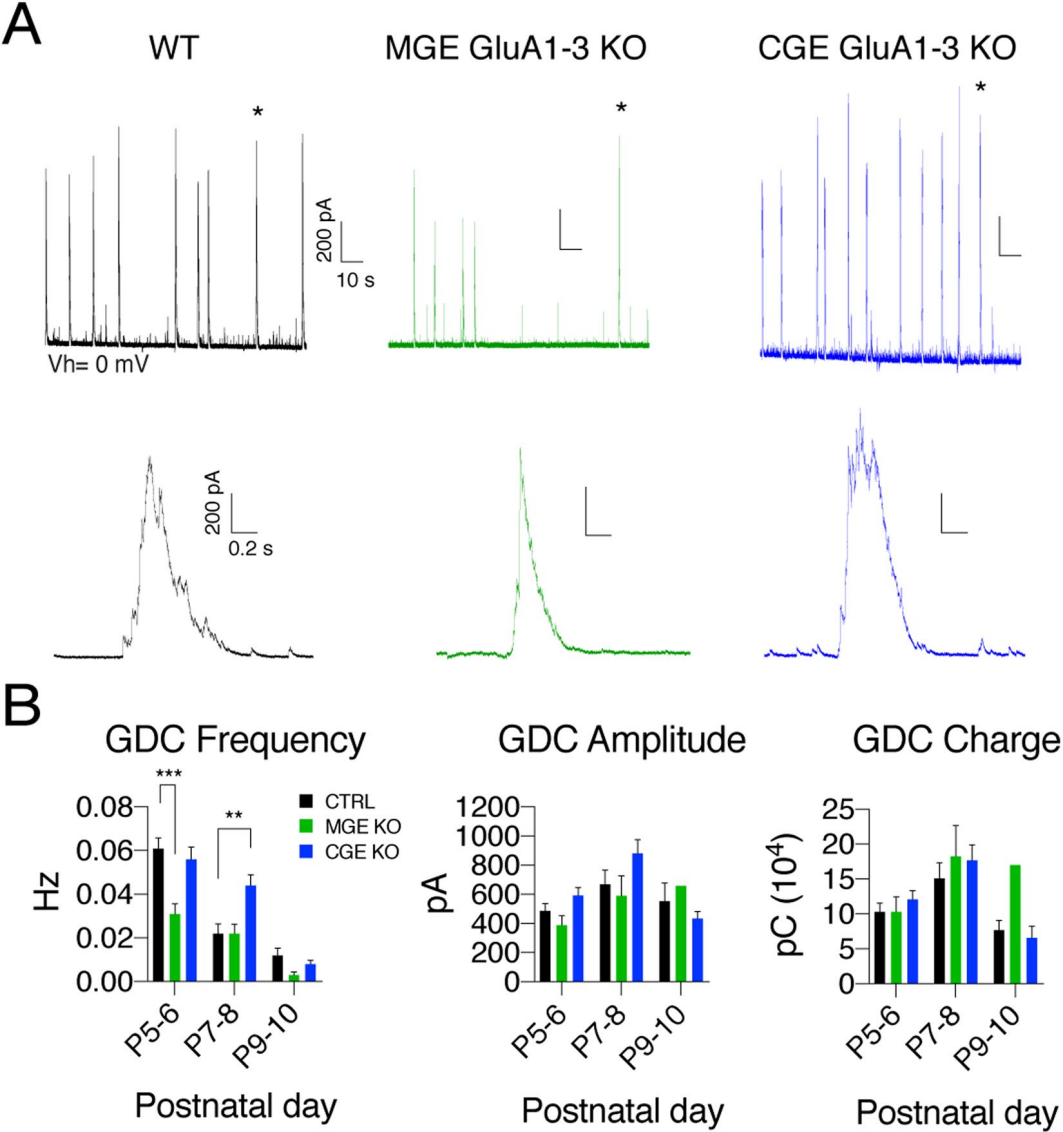
Excitatory synaptic input onto MGE interneurons is critical for GDC generation. GDCs recorded from P5-10 CA3 and CA1 pyramidal neurons of WT (black), MGE GluA1-3 KO (green) and CGE GluA1-3 KO (blue). Upper traces show 120 sec of recording and lower traces show the enlarged image of a 2 sec recording focusing on the representative single event marked by a * in the respective upper trace (**A**). Data were plotted and analyzed in consecutive two day periods to illustrate changes in GDC frequency, amplitude and charge transfer across a 6 day developmental window (**B**). **P < 0.005, ***P < 0.0005.

MGE-derived interneurons are crucial regulators of network activity in both early postnatal^14,16^ and adult hippocampus^17,18^. In a previous study using an optogenetic strategy, we demonstrated that MGE-derived inhibitory interneurons were essential for generation of the network oscillations seen early in development known as Giant depolarizing potentials (GDPs)^14^. GDPs are generated within the first two postnatal weeks and rely on the interplay between principal cells and interneuron-driven depolarizing GABA-mediated transmission^19,20^. Optical silencing of MGE- but not CGE-derived interneurons abolished ongoing GDPs, confirming an essential role for MGE-derived interneurons^14^. Whether spontaneous interneuron depolarization or excitatory synaptic drive recruits MGE-derived interneurons to generate GDPs is unexplored. Therefore, we next investigated the role of AMPAR-mediated synaptic recruitment of MGE-derived interneurons in GDP (subsequently referred to as GDCs since the experiments were performed in voltage clamp) activity in P5-10 developing hippocampus (Fig. 4). In recordings from both CA1 and CA3 pyramidal cells GDCs occurred at a significantly lower frequency in slices from P5-6 MGE GluA1-3 KOs compared to WT (WT, 0.061 ± 0.007 Hz, n = 46 cells from 8 animals; MGE KO, 0.030 ± 0.008, n = 23 cells from 4 animals. Two-way ANOVA F_interaction_(4,210) = 3.3, P = 0.01; F_age_(2,210) = 4.2, P = 0.02; F_genotype_(2,210) = 31.5, P < 0.0001; WT vs MGE KO, P < 0.0001; Dunnett’s multiple comparisons test) (Fig. 4A,B). Although the incidence of GDCs was reduced, neither the amplitude nor the underlying charge was altered in recordings from MGE GluA1-3 KOs compared to WT (WT, 459.2 ± 45.2 pA, n = 46 cells from 8 animals; MGE KO, 267.8 ± 36.7 pA, n = 23 cells from 4 animals; Two-way ANOVA F_interaction_(4,162) = 0.88, P = 0.5; F_age_(2,162) = 6.3, P = 0.002; F_genotype_(2,162) = 0.49, P = 0.6).

In our previous study^12^ elimination of GluA1-3 subunits from CGE-derived interneurons resulted in an ~95% decrease in sEPSCs onto CGE interneurons (a number comparable to that seen in MGE GluA1-3 KO, Fig. 2). To allow a direct comparison between the impact of AMPAR elimination in MGE- versus CGE-derived interneurons in GDC activity we took advantage of the 5HT3AR-Cre:Ai14:Gria1-3^f/f^ line which selectively eliminates AMPAR in CGE-derived interneurons (CGE GluA1-3 KO)^12^. CGE KO hippocampus showed no change in GDC frequency (WT, 0.061 ± 0.007 Hz, n = 46 cells from 8 animals; CGE KO, 0.056 ± 0.008, n = 34 cells from 6 animals; Two-way ANOVA, F_interaction_(4,210) = 3.3, P = 0.01; F_age_(2,210) = 4.2, P = 0.02; F_genotype_(2,210) = 31.5, P < 0.0001; WT vs CGE KO, P = 0.6; followed by Dunnett’s multiple comparisons test) and amplitude at P5-6 compared to WT (WT, 459.2 ± 45.2 pA, n = 46 cells from 8 animals; CGE KO, 593.6 ± 57.3 pA, n = 34 cells from 6 animals; Two-way ANOVA F_interaction_(4,162) = 0.88, P = 0.5; F_age_(2,162) = 6.3, P = 0.002; F_genotype_(2,162) = 0.49, P = 0.6) (Fig. 4A,B). Taken together these observations indicate that glutamatergic input onto MGE interneurons but not CGE interneurons is of primary importance for GDC generation in the early days of development.

GDPs reach a peak frequency around P5 and then decline during the next 5–6 days, a time frame consistent with the switch from depolarizing GABAergic drive to the conventional hyperpolarizing inhibition^21^. In P7-8 recordings GDC frequency was reduced compared to P5-6 but the difference in GDC frequency between WT and MGE KO groups disappeared (WT: 0.023 ± 0.004 Hz, n = 26 cells from 5 animals; MGE KO: 0.022 ± 0.004 Hz, n = 11 cells from 2 animals; CGE KO: 0.044 ± 0.005 Hz, n = 29 cells from 5 animals; Two-way ANOVA F_interaction_(4,210) = 3.3, P = 0.01; F_age_(2,210) = 4.2, P = 0.02; F_genotype_(2,210) = 31.5, P < 0.0001; WT vs MGE KO, P > 0.99; WT vs CGE KO, P = 0.004; followed by Dunnett’s multiple comparisons test) (Fig. 4B). Of interest, the GDC frequency of the CGE KO group was significantly higher than both WT and MGE KO at P7-8 suggesting that CGE interneurons may influence the time course of circuit maturation by an as yet unexplored mechanism. GDCs were virtually absent in recordings from P10 onwards in WT, MGE KO and CGE KO (P9-10, frequency: WT, 0.012 ± 0.003 Hz, n = 24 cells from 5 animals; MGE KO, 0.003 ± 0.001, n = 5 cells from 1 animal; CGE KO, 0.008 ± 0.002, n = 12 cells from 2 animals. Two-way ANOVA F_interaction_(4,210) = 3.3, P = 0.01; F_age_(2,210) = 4.2, P = 0.02; F_genotype_(2,210) = 31.5, P < 0.0001; amplitude: WT, 554 ± 125 pA, n = 8; MGE KO, 659 ± N/A pA, n = 1; CGE KO, 434 ± 49 pA, n = 9. Two-way ANOVA F_interaction_(4,162) = 0.88, P = 0.5; F_age_(2,162) = 6.3, P = 0.002; F_genotype_(2,162) = 0.49, P = 0.6, Fig. 4B), suggesting that the mechanisms that lead to the termination of GDC generation in the developing hippocampus are not dependent or perturbed by the presence or absence of AMPARs on inhibitory interneurons.

### A reduction in both feedforward and feedback inhibitory drive following loss of GluA1-3

**Figure 5:**
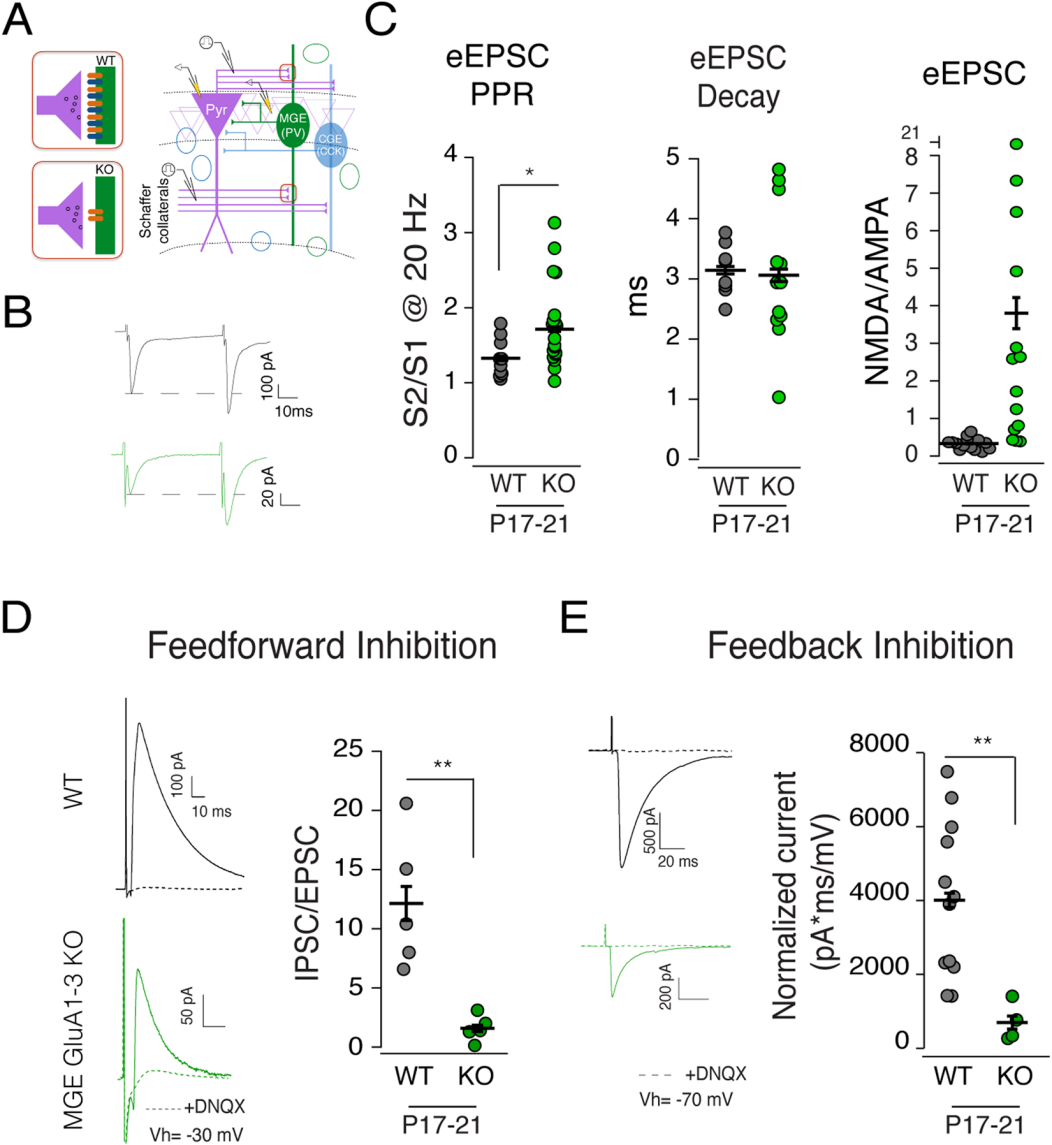
Loss of excitatory input onto MGE interneurons results in a reduction of both feedforward and feedback GABAergic input onto CA1 pyramidal neurons. (**A**) Schematic depicting the loss of AMPARs at the postsynaptic site of excitatory synapses onto KO MGE interneuron (left panels) and circuit arrangement for feedforward and feedback pathways in CA1 hippocampus (right panels). Stimulating electrodes were placed in the Schaffer collateral pathway to assay disynaptic feedforward inhibition provided to CA1 pyramidal neuron, and in alveus to antidromically activate the axons of pyramidal cells which then trigger disynaptic feedback inhibition. (**B**) Representative Schaffer collateral mediated EPSCs (eEPSC) recorded from WT and KO MGE interneurons were evoked by paired pulse stimuli (20 Hz) and show facilitation of the synaptic current. (**C**) Scatter plots show the group data for eEPSC paired pulse ratio (PPR), decay time constant and NMDA/AMPA ratio. The PPR of eEPSCs was higher in KO MGE interneurons compared to WT. eEPSC decay on average was similar between WT and KO but in KO it showed a wider range of values. NMDA/AMPA EPSC ratio was larger in KO compared to WT due to AMPAR loss at synapses. (**D**) As a consequence of the large reduction in excitatory recruitment of MGE interneurons disynaptic feedforward inhibition onto CA1 pyramidal neurons (monitored at a holding potential of −30mV. Outward IPSCs were normalized to the inward monosynaptic EPSCs) was reduced in the MGE GluA1-3 KO group. IPSC/EPSC ratios for all recordings are shown in the scatter plot for the group data. (**E**) Di-synaptic feedback inhibition was also reduced in MGE GluA1-3 KO CA1 hippocampi. Evoked IPSC amplitude was normalized to the slope of the population spike recorded from PCL. Normalized IPSC is shown in the scatter plot for the group data. The wildtype control dataset for both the feedforward and feedback inhibition experiments is replotted from our earlier study^12^ since all experiments were originally performed by interleaving recordings from wildtype, MGE-KO and CGE-KO animals. *P < 0.05, **P < 0.005.

The elimination of GluA1-3 made it difficult to reliably evoke Schaffer-collateral mediated synaptic events onto MGE-derived interneurons (Fig. 5A,B). When events *were* detected the kinetics of the residual effects did not differ from WT (Fig. 5B,C). In agreement with sEPSC data (Fig. 2), the eEPSC decay time constant was comparable across groups (WT: 3.14 ± 0.15 ms, n = 8 cells from 4 animals versus KO: 3.25 ± 0.38 ms, n = 15 cells from 6 animals, P17-21; P = 0.59, Mann-Whitney Test). As expected from the near elimination of the AMPAR-mediated component the NMDAR:AMPAR ratio was markedly increased compared to WT (WT: 0.33 ± 0.04, n = 14 cells from 10 animals versus KO: 3.81 ± 1.43, n = 14 cells from 5 animals; Fig. 5C). The EPSC paired pulse ratio (PPR) showed an unexpected increase in the KO population compared to WT (PPR_WT_, 1.32 ± 0.08, n = 10 cells from 4 animals versus PPR_KO_, 1.77 ± 0.12, n = 20 cells from 6 animals; P = 0.01, Mann-Whitney Test) (Fig. 5C), suggesting that elimination of AMPARs during synapse development may have direct influence over the establishment of the release probability of the presynaptic terminal. A result similar to our observations following elimination of NMDARs in CGE-derived interneurons^13^.

MGE-derived interneurons are major drivers of both feedforward and feedback inhibition onto pyramidal neurons^1,22–24^. To evaluate feedforward inhibitory drive onto CA1 pyramidal neurons, we next recorded from pyramidal neurons while stimulating Schaffer collateral afferents (Fig. 5A) to record monosynaptic EPSCs (detected as an inward current) and disynaptic IPSCs (outward currents) (Fig. 5D). Under this configuration we observed a dramatic reduction in the IPSC:EPSC ratio in MGE KO hippocampi compared to WT (IPSC/EPSC: WT, 12.13 ± 2.34, n = 5 cells from 3 animals; KO, 1.57 ± 0.63, n = 5 from 2 animals; P = 0.008, Mann Whitney Test) (Fig. 5D). Similarly, feedback inhibition elicited by local antidromic stimulation of CA1 pyramidal cell axons in the alveus to trigger disynaptic feedback input onto CA1 pyramidal cells (Fig. 5A) was decreased in the KO group compared to WT (Normalized IPSC: WT, 4007 ± 607 pA*ms/mV, n = 12 cells from 7 animals; KO, 698 ± 263 pA*ms/mV, n = 4 cells from 2 animals; P = 0.001, Mann Whitney Test) (Fig. 5E).

### Calcium permeable AMPAR expression is not required for postsynaptic NMDAR expression or maturation in MGE interneurons

**Figure 6:**
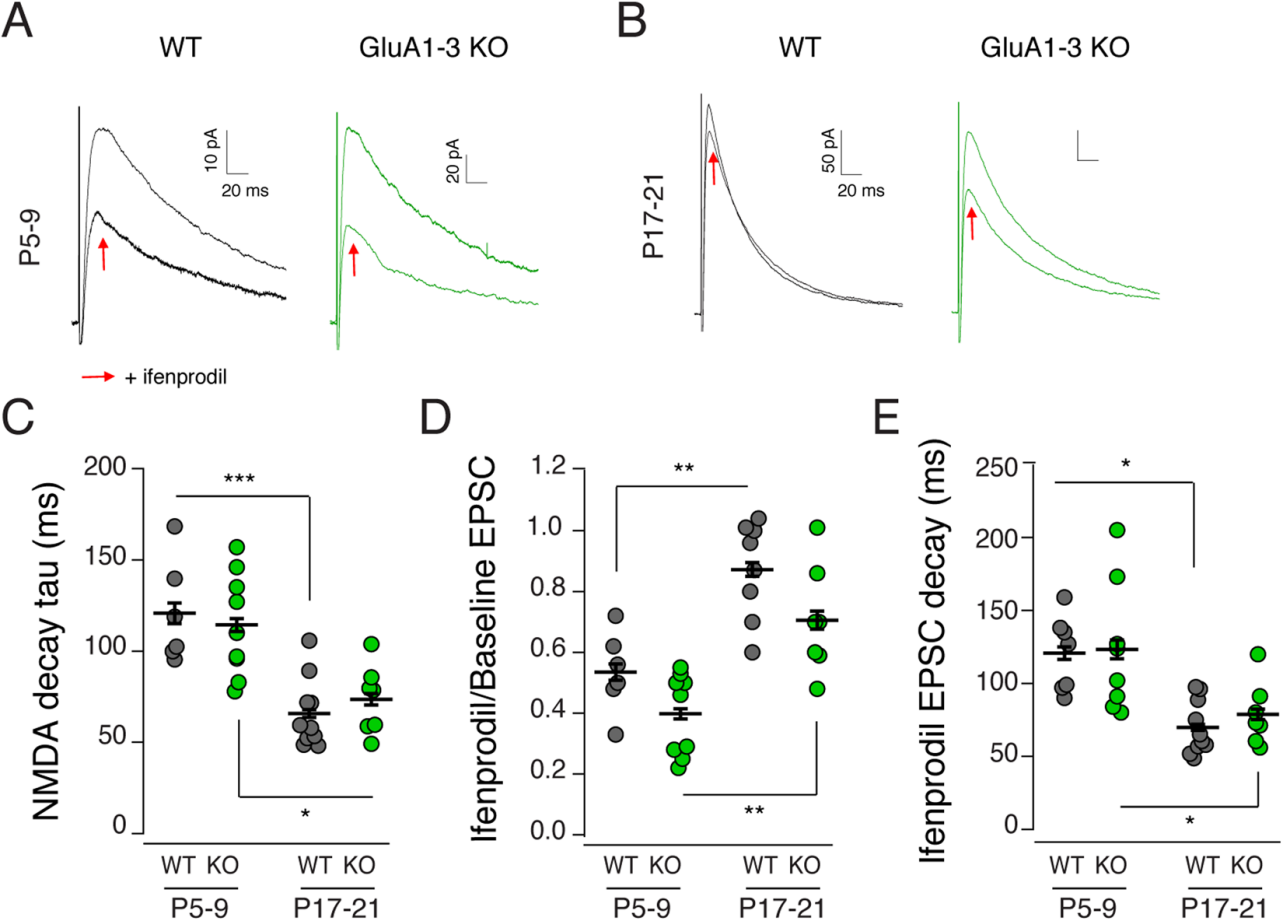
The GluN2B-to-2A developmental switch occurs independently of AMPARs. Schaffer collateral evoked NMDAR-mediated synaptic responses (Vhold =  + 50 mV) were recorded from WT (black) or GluA1-3 KO MGE (green) interneurons in CA1 hippocampi at P5-9 (**A**) and P17-21 (**B**) in the presence or absence of the GluN2B specific NMDAR antagonist ifenprodil (smaller traces indicated by red arrow) (all recordings done in the presence of picrotoxin (50uM) and DNQX (10uM)). The group data showed a significant decrease in decay tau (**C**) of NMDAR-mediated EPSCs between P5-9 to P17-21 in both WT and KO groups. NMDAR-mediated events became significantly less sensitive to ifenprodil from P5-9 to P17-21 (**D**,**E**). However, the loss of GluARs was without any major impact on the kinetic change or sensitivity of ifenprodil across the developmental window tested. *P < 0.05, **P < 0.005, ***P < 0.0005.

NMDARs on MGE-derived inhibitory interneurons possess a high GluN1/GluN2B content early in development, which then switches to GluN1/GluN2A in the second postnatal week^3^. In MGE-derived interneurons this GluN2B- GluN2A switch requires an elevation of intracellular Ca^2+^ thought to arise from Ca^2+^ entry through Ca^2+^-permeable AMPARs^3^. To determine whether this developmental subunit switch would proceed in the absence of AMPARs, we recorded NMDAR-mediated EPSCs (holding potential + 50 mV) from MGE-derived GluA1-3 KOs at both early postnatal (P5-9) and juvenile (P17-21) time points when GluN2B or GluN2A subunits predominate in WT. To determine the contribution of GluN2B containing NMDARs to evoked synaptic events, we took advantage of the NMDAR antagonist ifenprodil, which selectively blocks GluN2B-containing receptors. Schaffer-collateral evoked NMDAR EPSCs were similar between WT and KO recordings (Fig. 6A,B). At P5-9, the weighted decay time constant was similar between the two groups (WT; 124.8 ± 10.6 ms, n = 7 cells from 3 animals and KO 114.4 ± 9.4 ms, n = 9 cells from 3 animals; P = 0.85, Tukey *posthoc* test following ANOVA, F(3,31) = 13.70, P < 0.0001) (Fig. 6A,C) as was the magnitude of block by ifenprodil (WT: 0.53 ± 0.05 of control, n = 6 cells from 3 animals; KO: 0.40 ± 0.04 of control, n = 9 cells from 3 animals; Fig. 6A,D). By P17-21, the NMDAR decay time constant for both WT and KO groups was significantly faster than observed in P5-6 recordings (P17-21 WT: 65.9 ± 6.0 ms, n = 10 cells from 7 animals; KO: 73.4 ± 5.6 ms, n = 9 cells from 4 animals; WT P5-9 versus P17-21, P < 0.0001; KO P5-9 versus P17-21, P = 0.003, Tukey *posthoc* test following ANOVA, F(3,31) = 13.70, P < 0.0001) (Fig. 6A–C), consistent with the switch from predominantly GluN2B-containing to GluN2A-containing receptors. However, we failed to observe a significant difference between the decay time constants in WT and KO interneurons measured at P17-21 (P = 0.93) as well as ifenprodil sensitivity (NMDAR-mediated EPSC amplitude ifenprodil/baseline at P5-9 WT: 0.53 ± 0.05, n = 6; KO: 0.40 ± 0.04, n = 9; at P17-21 WT: 0.90 ± 0.06, n = 9; KO: 0.71 ± 0.07, n = 7; at P5-9 WT versus KO, P = 0.36; at P17-21 WT versus KO, P = 0.08; WT P5-9 versus P17-21, P = 0.001; KO P5-9 versus P17-21, P = 0.002, Tukey posthoc test following ANOVA, F(3,27) = 16.90, P < 0.0001) (Fig. 6) suggesting that the GluN2B- GluN2A switch proceeds as normal despite the absence of AMPARs.

## Discussion

Activity, including excitatory synaptic activity, is an important factor that regulates cell survival^12,25,26^, migration^27,28^, synapse number^12^ and the nature of cellular connectivity^13^ in the developing brain. Activity-dependent gene expression typically relies on transient elevations of intracellular Ca^2+^-entry arising through voltage gated Ca^2+^-channels. MGE-derived inhibitory interneurons, in addition to expressing voltage gated Ca^2+^-channels express GluA2-lacking, Ca^2+^-permeable AMPARs^3,5,29–32^, that may provide a pivotal alternative route for Ca^2+^-entry to link environment and intracellular regulation of activity driven gene expression and development in interneurons. The expression of AMPAR subunits, at time points well in advance of migration termination and the establishment of synaptic sites, is consistent with roles other than synaptic transmission for these receptors early in development. The Nkx2.1-Cre mouse line allowed us to eliminate GluA1,2 &3 AMPAR subunits specifically from MGE-derived interneurons early in their development. Surprisingly, although early removal of AMPARs resulted in many functional changes, their elimination had little impact on numbers or positioning of surviving cells. This is in sharp contrast to others who have shown that pharmacological intervention of AMPARs in developing neocortex and hippocampus leads to both interneuron migration and morphological defects^9–11^. The reason for these discrepant results is at present unclear but likely point to limitations in the use of pharmacological intervention to unequivocally determine the roles of specific glutamate receptors in interneuron migration and development.

Electrophysiological interrogation of MGE-derived interneurons in GluA1,2 &3 loss of function mice confirm the near absence of AMPAR-mediated sEPSCs (by 94% in neonatal, and by 90% in young adult). The low frequency (~5–10% of WT) of sEPSCs remaining in both age ranges tested might suggest either that the Cre/loxP dependent knock-out is not 100% penetrant^13^ or that the remaining events represent GluA4 homomeric AMPARs. GluA4 receptor expression is developmentally regulated primarily in PV-containing interneurons and does not occur until the second postnatal week of life^4,30,33^. The slight increase in frequency of remaining sEPSCs in recordings from the older age group is consistent with the postnatal upregulation of GluA4. However, the GluA4 subunit typically endows rapid kinetic properties to AMPARs^34,35^, and the events that remained did not possess more rapid decay time constants compared to their P5-9 counterparts (P5-9: 1.45 ± 0.07 ms, n = 5, versus 1.83 ± 0.05 ms, n = 8, P = 0.23, Mann-Whitney Test; Fig. 2C).

Unlike principal neurons which receive excitatory synaptic input only onto dendritic locations, particularly spines, excitatory synapses onto interneurons are found on both soma and smooth (non-spiny) dendrites^6^. Although few studies have analyzed the total number of excitatory synapses onto particular interneuron subtypes, rat PV-containing interneurons receive 5-10x more excitatory inputs than their CGE-derived counterparts^36,37^. The density of excitatory inputs onto PV-containing interneuron dendrites is almost 4-fold greater than seen on the soma, and it is unclear whether distinct afferents target somatic versus dendritic locations. In hippocampus and cerebellum, recordings from the dendrites of principal neurons show larger EPSPs with slower kinetics compared to events recorded at somatic locations^38–41^. These reports highlight that the properties of excitatory synapses distributed through soma and dendrites of neurons vary proportionally with the distance from the action potential initiation site to coordinate the input-output relationship of the neuron. It is unclear whether such an arrangement exists in interneurons, which are comparatively electrotonically compact. Interestingly, in our study, excitatory synapses were redistributed away from dendritic sites toward somatic locations in the GluA1,2&3 KO, which may be a compensation for the lack of total excitation being received by the cell. Synapse relocation toward somatic sites coupled to the observed changes in paired pulse ratio and probability of release may act to increase the likelihood of action potential firing in KO neurons in the face of reduced afferent excitatory drive.

It is well established that MGE-derived interneurons are major participants of oscillatory activity in early postnatal hippocampus^1,14,16^. We have previously shown that optogenetic inactivation of MGE interneuron activity, but not CGE interneurons eliminates ongoing GDP activity^14^. The role played by synaptic AMPARs on interneurons in generating GDPs however has not been explored. In this study the frequency and amplitude of sEPSCs received by neonatal MGE was higher (2.15 Hz, ~40pA), than we observed onto neonatal CGE interneurons at an equivalent (0.67 Hz, ~30pA)^12^ suggesting a more prominent role for excitatory drive onto MGE-derived interneurons at this age. Consistent with this observation GDC frequency was reduced by ~50% in MGE KO but unchanged in CGE KO hippocampi at P5-6 confirming that excitatory input onto MGE- but not CGE-derived interneurons is a major driver of synchronized network activity in neonatal hippocampus. Interestingly, the time course of developmental downregulation of GDC activity was similar in both WT and MGE-KO suggesting a diminished role for glutamate receptor drive onto MGE-derived interneurons as this developmental epoch progresses.

In the present study the I/E ratio of feedforward inhibitory drive was reduced by >80% following AMPAR elimination, consistent with a large reduction in excitatory drive onto MGE-derived interneurons. In our previous study, the loss of AMPARs from synapses onto CGE-derived interneurons reduced the I/E ratio by a similar amount (~76%)^12^. It is difficult to reconcile these two numbers since they should not be treated as only representing the arithmetic subtraction of MGE (e.g. PV-containing) versus CGE- (e.g. CCK-containing) derived interneuron contributions to inhibitory tone^42^ for a number of reasons. In both of these studies although the primary change was a loss of excitatory drive onto the MGE- (this study) or CGE-inhibitory^12^ subpopulations, the numerous other changes observed also likely contribute to the overall recruitment of inhibitory drive. Specifically across these two studies we have also shown that elimination of AMPARs changes cell number and cell subpopulation survival, alterations in cell morphology and axonal arborization, changes in presynaptic release probability, and anatomical redistribution of synaptic sites across the somatic-dendritic axis, all of which will impact the recruitment of the remaining interneuron populations, complicating a meaningful direct comparison of the magnitude of inhibition recruit in the two studies.

Recent reports of de novo mutations in glutamate receptor subunits (e.g., Gria in five individuals with learning disabilities and autism^43–46^) points to a link between glutamate receptors and channelopathies that lead to neurodevelopmental disorders. Similarly, somatic mutations during embryonic development are recognized as an underpinning factor for neurodevelopmental disorders^47,48^. However, these recent insights have tended to focus on the large pyramidal neuron population of the hippocampus and cortex. As interneurons derive from progenitors in distinct embryonic brain regions^49,50^ (i.e., CGE- and MGE-derived interneuron subgroups), it is reasonable to hypothesize that they may differentially accumulate numerous somatic mutations that would have multiple disparate consequences for circuit activity and brain function. Here, we establish a role for AMPARs in synaptic maturation in MGE-derived interneurons that contrasts with previous reports of AMPAR subunit deletions in pyramidal neurons^51^, and CGE-derived inhibitory interneurons^12^. Taken together these reports suggest that genetic permutations depending on what progenitor pool they occur in will result in unique morphological, physiological and behavioral outcomes. Since we have the opportunity to make specific base editing on the genome with CRISPR system, we can now study the relationship between identified/proposed mutations and malfunctions of glutamate receptors in animal models in a cell type specific manner.

## Materials and Methods

All animal studies have been approved by, and all methods were performed in accordance with the guidelines and regulations set by the National Institutes of Health’s Institutional Animal Care and Use Committee (IACUC).

### Animals

An Nkx2.1-Cre driver line (C57BL/6J-Tg(Nkx2-1-cre)2Sand/J; cat # 008661 Jackson Laboratory), a tdTomato reporter line (Ai14 (B6;129S6-*Gt(ROSA)26Sor*^*tm14(CAG-tdTomato)Hze*^/J; cat # 007914 Jackson Laboratory), and a floxed AMPAR subunit line (Gria1-3^fl/fl^ ^12,51–53^ obtained from Dr. W Lu (NINDS, NIH)) were crossed to generate the control mouse line (Nkx2.1-Cre:Ai14, WT^13^), and the triple knockout mouse line (Nkx2.1-Cre:Ai14:Gria1-3^fl/fl^, MGE GluA1-3 KO). A 5HT3R-Cre driver line (Htr3A BAC-Cre mouse line (founder line NO152) (obtained from Dr. C. Gerfen NIMH, NIH)) was used to generate the CGE-derived interneuron GluA1-3 KO line^12^. Both male and female mice were used for experiments.

### Histology

#### Tissue collection

For cell number-, and for synapse-quantification mice at P21, and P35-40 were used respectively as described in Akgul, *et al*.^12^ tdTomato fluorescence was used to identify MGE-derived interneurons in hippocampus of 50 μm coronal sections prepared from the dissected brains, without antibody labelling.

#### Recombinant virus production

An AAV vector carrying the coding sequence of PSD-95 with an enhanced green fluorescent protein (EGFP) gene at the C terminus was cloned as described in Akgul and Wollmuth^54^. A FLEX-rev-PSD-95-EGFP_AAV was produced by the University of North Carolina Gene Therapy Program Vector Core.

*Stereotaxic injections into hippocampus* were performed as described in Akgul, *et al*.^12^ Two control and two KO mice (P19-22) were anesthetized with isofluorane and 0.4 μl of AAV was injected at a rate of 0.1 μl/min into the hippocampus at to the following stereotaxic coordinates: −2.0 mm anterior to lambda; 2.0 mm lateral from the midline; 1.5 mm down from the dural surface, using a stereotaxic apparatus (Stoelting, Wood Dale, IL, USA). Mice were sacrificed at least two weeks after viral injection.

*Imaging and quantification* were performed as described in Akgul, *et al*.^12^ For cell number quantification, 4–6 sections of 50 μm thickness were used per animal. Sections were collected using systematic-random sampling^15^, with 5 animals per group. A Zeiss LSM Confocal microscope within the Microscopy and Imaging Core, NICHD, was used to capture fluorescent images of the hippocampi with a 10X objective. For synapse number quantification 2 mouse brains from each group (WT and KO) were dissected 14–15 days post-injection. 4–5 mounted sections without antibody labelling were imaged using a Zeiss LSM 880 with Airyscan at the Microscopy and Imaging Core, NICHD, with 63X objective. The EGFP signal on tdTomato positive dendrites and soma were quantified by using Spot Detection tool of *IMARIS* software version 8 (Bitplane, Zurich, Switzerland).

### Electrophysiology

Acute slices were prepared from mice between postnatal day 5–10 (P5-10, age group for GDC recordings), P5-9 (neonatal) and P17-21 (juvenile) for whole cell patch clamp electrophysiology as described in^12^.

#### Solutions

The artificial cerebrospinal fluid (ACSF) bath solution (in mM): 130 NaCl, 3.5 KCl, 10 D-glucose, 24 NaHCO_3_, 1.25 NaH_2_PO_4_, 2.5 CaCl_2_, 1.5 MgCl_2_, (saturated with 95% O_2_/5% CO_2_ under all conditions, pH 7.4). For feedforward inhibition experiments, CaCl_2_ was increased to 4.5 mM and the GABA_B_ receptor antagonist, CGP55845 (1 μM) was included.

Intracellular solutions (in mM): (1) 130 CsCl, 8.5 NaCl, 5 HEPES, 4 MgCl_2_, 4 Na_2_ATP, 0.3 NaGTP and 5 QX-314 (Tocris), pH 7.2–7.3 for sIPSC and eIPSC recordings; (2) 130 Cs-gluconate, 0.6 EGTA, 10 Bapta, 10 HEPES, 2 MgCl_2_, 2 Na_2_ATP, 0.3 NaGTP and 6 KCl, pH 7.2–7.3 for sEPSC at −70 mV, eEPSC at −70 mV and GDC at 0 mV recordings; and (3) 137 CsCH_3_SO_4_, 4.5 NaCl, 10 HEPES, 4 MgATP, 0.3 NaGTP, 5 QX-314, pH 7.2–7.3, for both eEPSC and eIPSC in feedforward inhibition experiments.

Picrotoxin (50 μM) was also used to isolate sEPSCs and eEPSCs. NMDAR-mediated EPSCs were isolated by inclusion of the AMPAR antagonist DNQX (10 μM), in addition to picrotoxin, and confirmed using the NMDAR antagonist DL-APV (100 μM). GDC’s were recorded with no blockers in the bath. They were completely blocked by picrotoxin (data not shown). Multipeak, outward GDC events were detected using built-in event detection protocol with a set threshold of 100 pA amplitude and 100 ms duration in Clampfit version 10.7. The wildtype control dataset for both the feedforward and feedback inhibition experiments is replotted from our earlier study^12^ since all experiments were originally performed by interleaving recordings from wildtype, MGE-KO and CGE-KO animals.

All data were filtered at 3 kHz and acquired at a sampling rate of 10 kHz using pClamp9.2 (Molecular Devices, Sunnyvale, CA, USA).

## Data Availability

The datasets generated and/or analysed during the current study are available from the corresponding author on reasonable request. Data were kept and analyzed in Excel or IGOR Pro (Wavemetrics) version 6.03 or GraphPad Prism version 8.2. Data were presented as mean ± SEM.

## References

[1] Pelkey KA (2017). Hippocampal GABAergic Inhibitory Interneurons. Physiol. Rev..

[2] Tricoire L (2011). A blueprint for the spatiotemporal origins of mouse hippocampal interneuron diversity. J. Neurosci..

[3] Matta JA (2013). Developmental origin dictates interneuron AMPA and NMDA receptor subunit composition and plasticity. Nat. Neurosci..

[4] Pelkey KA (2015). Pentraxins coordinate excitatory synapse maturation and circuit integration of parvalbumin interneurons. Neuron.

[5] Akgul G, McBain CJ (2016). Diverse roles for ionotropic glutamate receptors on inhibitory interneurons in developing and adult brain. J. Physiol..

[6] Freund, T. F. & Buzsaki, G. Interneurons of the hippocampus. *Hippocampus* **6**, 347-470, 10.1002/(SICI)1098-1063(1996)6:4<347::AID-HIPO1>3.0.CO;2-I (1996). 10.1002/(SICI)1098-1063(1996)6:4<347::AID-HIPO1>3.0.CO;2-I 8915675

[7] Monyer H, Burnashev N, Laurie DJ, Sakmann B, Seeburg PH (1994). Developmental and regional expression in the rat brain and functional properties of four NMDA receptors. Neuron.

[8] Monyer H, Seeburg PH, Wisden W (1991). Glutamate-operated channels: developmentally early and mature forms arise by alternative splicing. Neuron.

[9] Manent JB, Represa A (2007). Neurotransmitters and brain maturation: early paracrine actions of GABA and glutamate modulate neuronal migration. Neuroscientist: a Rev. J. bringing neurobiology, Neurol. psychiatry.

[10] Manent JB, Jorquera I, Ben-Ari Y, Aniksztejn L, Represa A (2006). Glutamate acting on AMPA but not NMDA receptors modulates the migration of hippocampal interneurons. J. Neurosci..

[11] Poluch, S. *et al*. AMPA receptor activation leads to neurite retraction in tangentially migrating neurons in the intermediate zone of the embryonic rat neocortex. *Journal of neuroscience research* **63**, 35-44, 10.1002/1097-4547(20010101)63:1<35::AID-JNR5>3.0.CO;2-1 (2001). 10.1002/1097-4547(20010101)63:1<35::AID-JNR5>3.0.CO;2-1 11169612

[12] Akgul G, Abebe D, Yuan XQ, Auville K, McBain CJ (2019). The Role of AMPARs in the Maturation and Integration of Caudal Ganglionic Eminence-Derived Interneurons into Developing Hippocampal Microcircuits. Sci. Rep..

[13] Chittajallu R (2017). Afferent specific role of NMDA receptors for the circuit integration of hippocampal neurogliaform cells. Nat. Commun..

[14] Wester JC, McBain CJ (2016). Interneurons Differentially Contribute to Spontaneous Network Activity in the Developing Hippocampus Dependent on Their Embryonic Lineage. J. Neurosci..

[15] Akgul G, Wollmuth LP (2010). Expression pattern of membrane-associated guanylate kinases in interneurons of the visual cortex. J. Comp. Neurol..

[16] Flossmann T (2019). Somatostatin Interneurons Promote Neuronal Synchrony in the Neonatal Hippocampus. Cell Rep..

[17] Cossart R (2011). The maturation of cortical interneuron diversity: how multiple developmental journeys shape the emergence of proper network function. Curr. Opin. Neurobiol..

[18] Allene C (2012). Dynamic changes in interneuron morphophysiological properties mark the maturation of hippocampal network activity. J. Neurosci..

[19] Ben-Ari Y (2014). The GABA excitatory/inhibitory developmental sequence: a personal journey. Neurosci..

[20] Pelkey KA (2016). Pentraxins Coordinate Excitatory Synapse Maturation and Circuit Integration of Parvalbumin Interneurons. Neuron.

[21] Ben-Ari Y, Cherubini E, Corradetti R, Gaiarsa JL (1989). Giant synaptic potentials in immature rat CA3 hippocampal neurones. J. Physiol..

[22] Pouille F, Scanziani M (2001). Enforcement of temporal fidelity in pyramidal cells by somatic feed-forward inhibition. Sci..

[23] Yuan, M. *et al*. Somatostatin-positive interneurons in the dentate gyrus of mice provide local- and long-range septal synaptic inhibition. *eLife* **6**, 10.7554/eLife.21105 (2017). 10.7554/eLife.21105 PMC5395294 28368242

[24] Bartos M, Alle H, Vida I (2011). Role of microcircuit structure and input integration in hippocampal interneuron recruitment and plasticity. Neuropharmacol..

[25] Denaxa M (2018). Modulation of Apoptosis Controls Inhibitory Interneuron Number in the Cortex. Cell Rep..

[26] Priya R (2018). Activity Regulates Cell Death within Cortical Interneurons through a Calcineurin-Dependent Mechanism. Cell Rep..

[27] Denaxa M (2012). Maturation-promoting activity of SATB1 in MGE-derived cortical interneurons. Cell Rep..

[28] Karayannis T, De Marco Garcia NV, Fishell GJ (2012). Functional adaptation of cortical interneurons to attenuated activity is subtype-specific. Front. Neural Circuits.

[29] Fuchs EC (2007). Recruitment of parvalbumin-positive interneurons determines hippocampal function and associated behavior. Neuron.

[30] Geiger JR (1995). Relative abundance of subunit mRNAs determines gating and Ca2+ permeability of AMPA receptors in principal neurons and interneurons in rat CNS. Neuron.

[31] Catania MV, Tolle TR, Monyer H (1995). Differential expression of AMPA receptor subunits in NOS-positive neurons of cortex, striatum, and hippocampus. J. Neurosci..

[32] Paul A (2017). Transcriptional Architecture of Synaptic Communication Delineates GABAergic Neuron Identity. Cell.

[33] Yamasaki M (2016). TARP gamma-2 and gamma-8 Differentially Control AMPAR Density Across Schaffer Collateral/Commissural Synapses in the Hippocampal CA1 Area. J. Neurosci..

[34] Bochet P (1994). Subunit composition at the single-cell level explains functional properties of a glutamate-gated channel. Neuron.

[35] Swanson GT, Kamboj SK, Cull-Candy SG (1997). Single-channel properties of recombinant AMPA receptors depend on RNA editing, splice variation, and subunit composition. J. Neurosci..

[36] Gulyas AI, Megias M, Emri Z, Freund TF (1999). Total number and ratio of excitatory and inhibitory synapses converging onto single interneurons of different types in the CA1 area of the rat hippocampus. J. Neurosci..

[37] Matyas F, Freund TF, Gulyas AI (2004). Convergence of excitatory and inhibitory inputs onto CCK-containing basket cells in the CA1 area of the rat hippocampus. Eur. J. Neurosci..

[38] Nevian T, Larkum ME, Polsky A, Schiller J (2007). Properties of basal dendrites of layer 5 pyramidal neurons: a direct patch-clamp recording study. Nat. Neurosci..

[39] Winters BD, Jin SX, Ledford KR, Golding NL (2017). Amplitude Normalization of Dendritic EPSPs at the Soma of Binaural Coincidence Detector Neurons of the Medial Superior Olive. J. Neurosci..

[40] Williams SH, Johnston D (1991). Kinetic properties of two anatomically distinct excitatory synapses in hippocampal CA3 pyramidal neurons. J. Neurophysiol..

[41] Tran-Van-Minh A, Abrahamsson T, Cathala L, DiGregorio DA (2016). Differential Dendritic Integration of Synaptic Potentials and Calcium in Cerebellar Interneurons. Neuron.

[42] Basu J (2013). A cortico-hippocampal learning rule shapes inhibitory microcircuit activity to enhance hippocampal information flow. Neuron.

[43] Geisheker MR (2017). Hotspots of missense mutation identify neurodevelopmental disorder genes and functional domains. Nat. Neurosci..

[44] Li J (2019). De Novo GRIN Variants in NMDA Receptor M2 Channel Pore-Forming Loop Are Associated with Neurological Diseases. Hum. Mutat..

[45] XiangWei W, Jiang Y, Yuan H (2018). De Novo Mutations and Rare Variants Occurring in NMDA Receptors. Curr. Opin. Physiol..

[46] Hamdan FF (2011). Excess of de novo deleterious mutations in genes associated with glutamatergic systems in nonsyndromic intellectual disability. Am. J. Hum. Genet..

[47] McConnell, M. J. *et al*. Intersection of diverse neuronal genomes and neuropsychiatric disease: The Brain Somatic Mosaicism Network. *Science* **356**, 10.1126/science.aal1641 (2017). 10.1126/science.aal1641 PMC5558435 28450582

[48] D’Gama AM, Walsh CA (2018). Somatic mosaicism and neurodevelopmental disease. Nat. Neurosci..

[49] Erickson RP (2016). The importance of de novo mutations for pediatric neurological disease–It is not all in utero or birth trauma. Mutat. Res. Rev. Mutat Res.

[50] Poduri A, Evrony GD, Cai X, Walsh CA (2013). Somatic mutation, genomic variation, and neurological disease. Sci..

[51] Lu W (2009). Subunit composition of synaptic AMPA receptors revealed by a single-cell genetic approach. Neuron.

[52] Engblom D (2008). Glutamate receptors on dopamine neurons control the persistence of cocaine seeking. Neuron.

[53] Shimshek DR (2005). Enhanced odor discrimination and impaired olfactory memory by spatially controlled switch of AMPA receptors. PLoS Biol..

[54] Akgul G, Wollmuth LP (2013). Synapse-associated protein 97 regulates the membrane properties of fast-spiking parvalbumin interneurons in the visual cortex. J. Neurosci..

